# Neurocognitive Systems Related to Real-World Prospective Memory

**DOI:** 10.1371/journal.pone.0013304

**Published:** 2010-10-08

**Authors:** Grégoria Kalpouzos, Johan Eriksson, Daniel Sjölie, Jonas Molin, Lars Nyberg

**Affiliations:** 1 Physiology Section, Department of Integrative Medical Biology, Umeå University, Umeå, Sweden; 2 Umeå Center for Functional Brain Imaging, Umeå, Sweden; 3 Department of Computing Science, Umeå University, Umeå, Sweden; 4 Diagnostic Radiology, Department of Radiation Sciences, Umeå University, Umeå, Sweden; Indiana University, United States of America

## Abstract

**Background:**

Prospective memory (PM) denotes the ability to remember to perform actions in the future. It has been argued that standard laboratory paradigms fail to capture core aspects of PM.

**Methodology/Principal Findings:**

We combined functional MRI, virtual reality, eye-tracking and verbal reports to explore the dynamic allocation of neurocognitive processes during a naturalistic PM task where individuals performed errands in a realistic model of their residential town. Based on eye movement data and verbal reports, we modeled PM as an iterative loop of five sustained and transient phases: intention maintenance before target detection (TD), TD, intention maintenance after TD, action, and switching, the latter representing the activation of a new intention in mind. The fMRI analyses revealed continuous engagement of a top-down fronto-parietal network throughout the entire task, likely subserving goal maintenance in mind. In addition, a shift was observed from a perceptual (occipital) system while searching for places to go, to a mnemonic (temporo-parietal, fronto-hippocampal) system for remembering what actions to perform after TD. Updating of the top-down fronto-parietal network occurred at both TD and switching, the latter likely also being characterized by frontopolar activity.

**Conclusion/Significance:**

Taken together, these findings show how brain systems complementary interact during real-world PM, and support a more complete model of PM that can be applied to naturalistic PM tasks and that we named PROspective MEmory DYnamic (PROMEDY) model because of its dynamics on both multi-phase iteration and the interactions of distinct neurocognitive networks.

## Introduction

Neuroimaging studies have yielded much information about task-induced functional brain changes in various domains of cognition, but this has almost exclusively been accomplished by using laboratory paradigms that hardly capture complex real-life behavior. Here, our objective was to study the dynamic allocation of neurocognitive processes during a naturalistic human activity called upon in everyday life: prospective memory (PM). PM refers to the ability to remember to perform previously planned actions in the future, such as doing errands [1]. PM requires multiple cognitive processes, such as attention, executive functions, and retrospective memory [2], [3]. PM has traditionally been described as consisting of successive phases or components such as formation of an intention – retention interval – recognition of the retrieval cue (e.g., the optician store) – remembering that something has to be done (*prospective* component *per se* of PM, e.g. something has to be done in relation to the optician store) – remembering what to do (*retrospective* component of PM, e.g., check out the price of a specific pair of glasses) – compliance [3]–[5]. However, a reduced number of components have usually been studied in behavioral experiments (e.g., prospective and retrospective components) [6], as well as in neuroimaging protocols (e.g., cue identification and intention retrieval) [7]. In contrast to behavioral studies [8], to date, only artificial computer-based laboratory tasks have been used to reveal brain areas involved in PM. Moreover, most of these tasks focused on the role of the frontopolar cortex in attentional processes, which has been suggested to be a key component of PM [5], [7], [9]–[14]. Recent methodological advances make it possible to overcome some limitations of standard laboratory tasks by using virtual reality (VR), where the subject is immersed in a virtual environment and interacts with it by acting on its elements. VR has been successfully used in combination with fMRI in a handful of experiments to address questions regarding spatial navigation and episodic memory [15], [16], and a few behavioral studies have used VR to examine PM [17].

We combined fMRI and a VR model that simulated the center of the residential town of the subjects and asked them to perform real-world errands within this environment. Each errand was conceptualized as one PM task and the subjects were free to solve a series of tasks within distinct routes in the order they found appropriate. This more open and thereby more realistic environment of the VR model comes with the cost of making it difficult to distinguish between various PM components and processes. The joint use of on-line eye-tracking and post-scan verbal reports has been proven helpful in overcoming this difficulty when using VR [18], [19]. The first step of the study was to characterize different PM phases in a detailed model. By contrast, in previous neuroimaging experiments a limited number of components were identified and investigated [7]. Secondly, while previous studies used VR to explore neural substrates of independent and specific mental events (e.g., “planning future movements with the vehicle”, “watching moving traffic in the environment”) [16], our main aim was to elaborate a more complete and systematic functional model of PM where different neurocognitive networks would be engaged. Such a model may better generalize to naturalistic prospective events that occur in everyday life.

## Materials and Methods

### Ethics Statement

The study was approved by the ethics committee of Umeå University. All participants gave written informed consent to participate.

### Subjects

Fourteen healthy subjects (mean age = 26.5±6.7 years old, 6 females), residents of the town of Umeå, Sweden, participated in this study. All but one were right-handed and all had normal or corrected-to-normal visual acuity. None of the subjects had a history of neurological or psychiatric illness. None of the enrolled individuals had any particular skill in navigation or orientation, besides knowing their home town. Thus, we aimed at generalizing to the general population rather than to some select population of experts (e.g. taxi drivers).

### General procedure

The day before scanning, the subjects were presented in random order pictures of the places (taken from the VR environment) together with their location on a map of the town in order to confirm their knowledge of downtown Umeå and the location of the places. They were not explicitly told that they had to memorize them. The instructions were to observe each place carefully because they might have to interact with it the day after in the virtual environment. They were then led to a dummy scanner and were familiarized with the apparatus (described below) for a few minutes with a prospective memory task (that was not part of the scanned session) for navigation and detection abilities. On the next day, they were installed in the MR device. They performed an encoding task in which they had to visualize themselves performing the actions presented one by one in random order (e.g., “Return a book to the library”). Then, following the T1-weighted MRI acquisition, they performed the PM-VR task. A verbal protocol took place immediately after the scanning session in another room (see below). Here we present the results of the PM-VR experiment.

### Apparatus

The virtual reality software system was based on Colosseum3D [20], developed at VRlab, Umeå University, and was specifically extended to handle the required devices and scenarios. The virtual environment was based on a 3D-model of downtown Umeå, built using Autodesk® 3ds Max® and populated with interactive objects to act as targets and triggers for the PM tasks. The system also included extensive logging of every event and the possibility to record and later play back an entire session including all available input.

The eye-tracking system was integrated into MR-compatible goggles (delivered by NordicNeuroLab, Bergen, Norway) mounted on the MR head coil. The system uses an infrared light source and a camera to produce a video signal (NTSC, 60 Hz, half frame) of the eye that is analyzed by a computer to get the positions of the pupil and corneal reflection. The gaze fixation point can then be calculated at each time point, based on this data and calibration data. Only movement of the right eye was recorded. Eye-tracking data was incorporated directly into the logged data from the VR environment and could therefore be projected onto the environment for later analyses.

A custom-made joystick (coupled optical 6-axis force-torque transducer) was used (right hand) to navigate in the virtual environment. The joystick enabled rotation and movement, separately or as a combined maneuver, in all directions allowed by the VR environment. A pistol-grip type of MR-compatible button was used (left hand) to trigger each task event.

### PM-VR task

**Figure 1:**
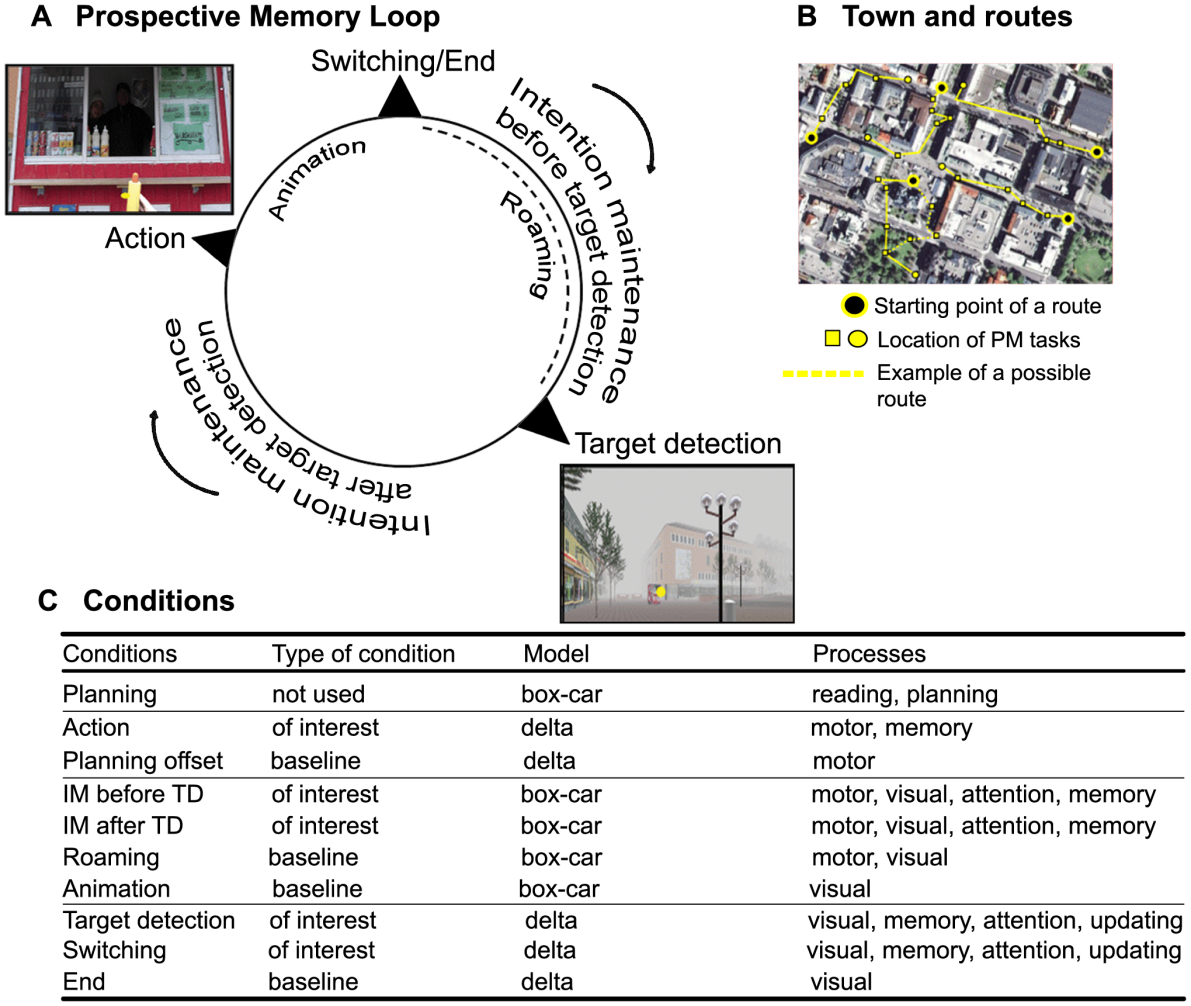
Illustration of the virtual reality PM task and the multi-phase iterative loop of PM. (A) Each PM task (e.g., buy a hotdog) was characterized as a loop composed of 5 phases: (1) Intention maintenance before target detection (TD): the subject is actively looking for the hotdog stand, (2) TD: the subject detects the stand (the yellow dot represents gaze fixation via the eye-tracking system), (3) Intention maintenance after TD: the subject is heading towards the stand with the intention of buying a hotdog, (4) Action: the subject presses the button to indicate that he or she buys a hotdog, (5) Switching: the task is terminated and the subject activates another intention in mind; “End” was used instead of switching at the end of the last task of a route performed by the subject. Note that “Roaming” replaced Intention maintenance before TD (phase 1) in cases where no intention was activated by the subject but was triggered by the perception of the target. Animation occurred when the subject pressed the button to indicate the execution of the task and displayed the action. (B) Showing of the five routes (depicted in yellow) that composed the task (zoom of Umeå center, Google™ Earth, version 5.0 beta, Google, Inc.). Each route contained 4 or 5 tasks to accomplish in the order the subjects found appropriate. For some tasks two targets were active in the virtual environment (e.g., two optician stores), the subjects being able to choose either target, resulting in other possible routes (e.g., dotted line) and greater flexibility of the PM task. (C) The table indicates the different conditions used in the present experiment and their characteristics. “Planning” occurred at the beginning of each route, where the instructions were shown to the subject, who in turn pressed a button (“Planning offset”) to start the route.

The PM task was divided into five routes (Fig. 1B) and overall there were 22 tasks to be performed, 4 or 5 per route. At the start of each route, instructions were displayed on the screen describing the tasks to be performed. For example, the instructions for the first route were as follows: “Your task is to go to Rådhustorget (*City Hall Square*) via Kungsgatan (*name of a street*) and do the following along the way:

- Check the price of Ray-Ban glasses at Synoptik or Synsam
- Look through the window of Ljungs ur (*jewelry store*)
- Throw a candy wrapper in the trashcan
- Pick up a copy of today's Metro (*free newspaper*)

Consider yourself as already having the items needed to complete the tasks. Press the button with the left index finger to move on”.

When the subject had read the tasks, he or she pressed the button of the pistol-grip, and was enabled to navigate in the VR environment using the joystick. When detecting a target (e.g., a phone booth), he or she was instructed to navigate towards it and press the button when reaching the target, representing the action to be performed. An animation showing the action was then automatically displayed (e.g., making a phone call). When the participant successfully performed all tasks of one route, an “end” text was displayed on the screen. Before the first route, in between the routes, and at the end of the VR task, a calibration procedure was completed to coordinate gaze and screen location. There was no time limit for the VR task. The subjects were free to solve the tasks in the order they found appropriate (the tasks were not presented in any particular order). If a subject met some difficulty and was unable to complete a route (e.g., he or she could not find a place, or forgot a PM task), the experimenters stopped the ongoing route and shifted to the next one (frequency <3% of all tasks for all subjects).

Directly after the fMRI session, the subjects completed a verbal report protocol session [18], [21]. They were installed in an adjacent room and first performed a number of “warm-up” exercises for “thinking aloud”, such as verbalizing arithmetic and geometric problem solutions. A movie of their own performance during scanning was then displayed on a screen. Their task was to describe their thoughts and actions while they were performing the PM task in as much detail as possible. The movie was paused or rewound when necessary to capture the full details of the reports. The experimenter interrupted the subject's reports as little as possible, only asking follow-up questions to clarify statements when necessary. Importantly, in order not to influence their reports, the movie did not contain their eye-tracking data. The verbal material was recorded and later synchronized with the VR performance and eye-movements recordings in order to extract the onsets of interest. The verbal report protocol served two purposes: to identify the subject's intentions as they proceeded through the PM tasks and to verify the accuracy of the fixation as representing detection of the target (see Movie S1).

### Identification of 5 PM phases

We identified five successive time-periods of interest, corresponding to different PM phases (Fig. 1). The *intention maintenance* phase was defined as the time from when a subject made the decision to perform a given action until the effective realization of that action. The detailed post-experiment reports allowed us to split this phase into two distinct phases separated by target detection (TD), since for most of the PM tasks active intention was not triggered by the perception of the target but was self-initiated during the period preceding TD (Table S1, and Movie S1 for an example of each occurrence). Thus, there was a first intention maintenance phase which lasted from the end of the previous task until TD, and a second intention maintenance phase which lasted from TD until action. While the presence of a delay before TD has previously been considered in neuroimaging PM studies [7], a maintenance phase between TD and action has not, despite the fact that such occurrences are typical in real-world situations. *TD* itself, which corresponds to the retrieval of the prospective component of PM (remembering that something has to be done in relation to a specific target), was defined as the time-point when the gaze was positioned on the target for the first time. These time-points were confirmed by the subjects in their verbal reports as constituting the times when they did recognize the targets as being prospective cues. *Action*, corresponding to the retrieval of the retrospective component of PM (i.e., what has to be done in relation to the target), was defined as the time when the subject pressed a button to indicate that he or she was performing the task. Finally, the *switching* phase was defined as the time-point when one task was finished and the subject had to switch focus from the just executed task and activate a new intention. Taken together, PM processing was characterized in terms of iterative loops consisting of the five successive phases, allowing the activation of the PM tasks in a continuous manner. Other phases that were not related to any active prospective memory processing were used as baselines in the neuroimaging analyses. Theses phases are described in the statistical analyses section below and in Fig. 1A and 1C.

### Neuroimaging procedure

The current fMRI study was carried out on a Philips 3.0 tesla Achieva using an 8 channel SENSE head coil. For the functional scanning the following parameters were used: repetition time: 1512 ms for three subjects and 1500 ms for the remaining subjects (31 slices acquired), echo time: 30 ms, flip angle: 70 degrees, field of view: 22×22 cm, 64×64 matrix and 4.65 mm slice thickness. To avoid signals arising from progressive saturation, ten dummy scans were performed prior to image acquisition. The PM task was designed to be run within one session. However, 2 sessions were needed for 5 subjects because of the limited number of scans allowed per session by the scanner (1000) or because of minor technical problems that did not affect the experiment; this was smoothly done in-between 2 routes, and the second session started with a calibration procedure to coordinate gaze and screen location. Structural high-resolution T1 images were also acquired. For the T1-weighted images a 3D turbo field-echo sequence was used with the following parameters: repetition time: 10.5 ms, echo time: 5 ms, flip angle: 8 degrees, and field of view: 24×24 cm. 170 sagittal slices with a slice thickness of 1 mm were acquired in 336×332 matrices and reconstructed to 800×800 matrices. All images were sent to a PC and converted to Analyze format.

### Statistical analyses

Functional images were pre-processed and analyzed using SPM5 (Statistical Parametric Mapping, Wellcome Trust Centre for Neuroimaging, http://www.fil.ion.ucl.ac.uk/spm/) implemented in Matlab 7.6 (Mathworks Inc, MA, US). After correcting for differences in slice timing within each image volume, all images were realigned to the first image volume acquired, then normalized to standard anatomic space defined by the MNI atlas, and finally spatially smoothed using an 8.0-mm full-width at half-maximum Gaussian filter kernel.

The two “intention maintenance” phases of PM were modeled as a fixed response waveform (box-car), whereas “TD”, “action” and “switching” were modeled as delta functions. Different baselines were used to investigate the main effect of each variable of interest (see Fig. 1C). The two intention maintenance phases were contrasted with periods when the participants did not have any active intention in mind (“roaming”), which notably occurred instead of “intention maintenance before TD” as indicated in Fig. 1A (this controlled for hand movements with the joystick), as well as the “animation” phase after the action was done and where an automatic animation symbolizing this action was displayed (this controlled for visual processing, Fig. 1A). A conjunction analysis (conjunction null approach) [22] and a direct comparison between the two maintenance phases were performed. “Action” was contrasted with the “instructions' offset”, when the subjects were ready to start a route by pressing the button (this controlled for the button press occurring at action, Fig. 1C). Both “TD” and “switching” were contrasted with the “end” events (Fig. 1A and 1C), corresponding to the end of the last task of each route, where the subject did not have any remaining intention to activate and where an “End” text was displayed before a new calibration procedure. Since eye-tracking data was not available for 2 subjects, analysis of TD was performed on 12 subjects, as well as the direct comparisons between the two intention maintenance phases. Also, “roaming” data was not available for two other subjects. Consequently, the conjunction analysis was performed on 10 subjects. The data of all 14 subjects were included for the other contrasts (“action” versus “instructions' offset”, and “switching” versus “end”). All models were convolved with a “canonical” hemodynamic response function as implemented in SPM5. Covariates of no interest included the six realignment parameters to account for signal-changes related to inadvertent head motion. Single-subject statistical contrasts were set up using the general linear model and group data were analyzed with a random-effects model. Using the explicit masking option of SPM5, contrast images obtained at the subject-level analyses were masked so as to include only gray-matter voxels, using the binarized mean T1-weighted image of the subjects. Statistical parametric maps were generated voxel by voxel using *t* statistics to identify regions activated according to the model. Results were considered significant at p<.001 uncorrected for multiple comparisons. Also, based on anatomical pre-defined hypotheses (frontopolar cortex and medial temporal lobe (MTL)) as well as low number of subjects and events (predictive of low statistical power), we also used the lenient threshold of p<.005.

Results were displayed at p<.005 for illustrative purposes using Anatomist (BrainVISA/Anatomist; http://www.brainvisa.info) and MRIcron (http://www.sph.sc.edu/comd/rorden/mricron). Histogram plots showing magnitude of activations (beta values) were also displayed on figures together with brain maps. For that purpose, using an in-house program (DataZ), mean beta values were extracted for all subjects using a 5mm radius sphere around activation peaks of interest.

## Results

### Behavioral results

All 14 subjects accomplished almost all 22 errands. Three subjects forgot to perform one task, and four failed to find a target. Also, one subject performed the same task twice but at two different places. Table S1 displays further information regarding behavior and the number of occurrences taken into account for each condition subject by subject and time duration when appropriate. We discarded the data for one route for subject 1 and two routes for subject 2 due to imperfect understanding of the instructions. We also discarded from one to five “intention maintenance before TD” events due to the difficulty met by some subjects to find those specific targets. Behavioral analyses showed that for most of the PM tasks intention was self-initiated by the subjects rather than triggered by the perception of the targets. Indeed, the difference between the number of “full PM loops” (including self-initiated intention, i.e. with intention maintenance before TD) and the number of “half PM loops” (when the intention was triggered by the perception of the target, i.e. without any intention maintenance before TD) was significant [t-test t(22) = 10.03, *p*<.0001].

**Figure 2:**
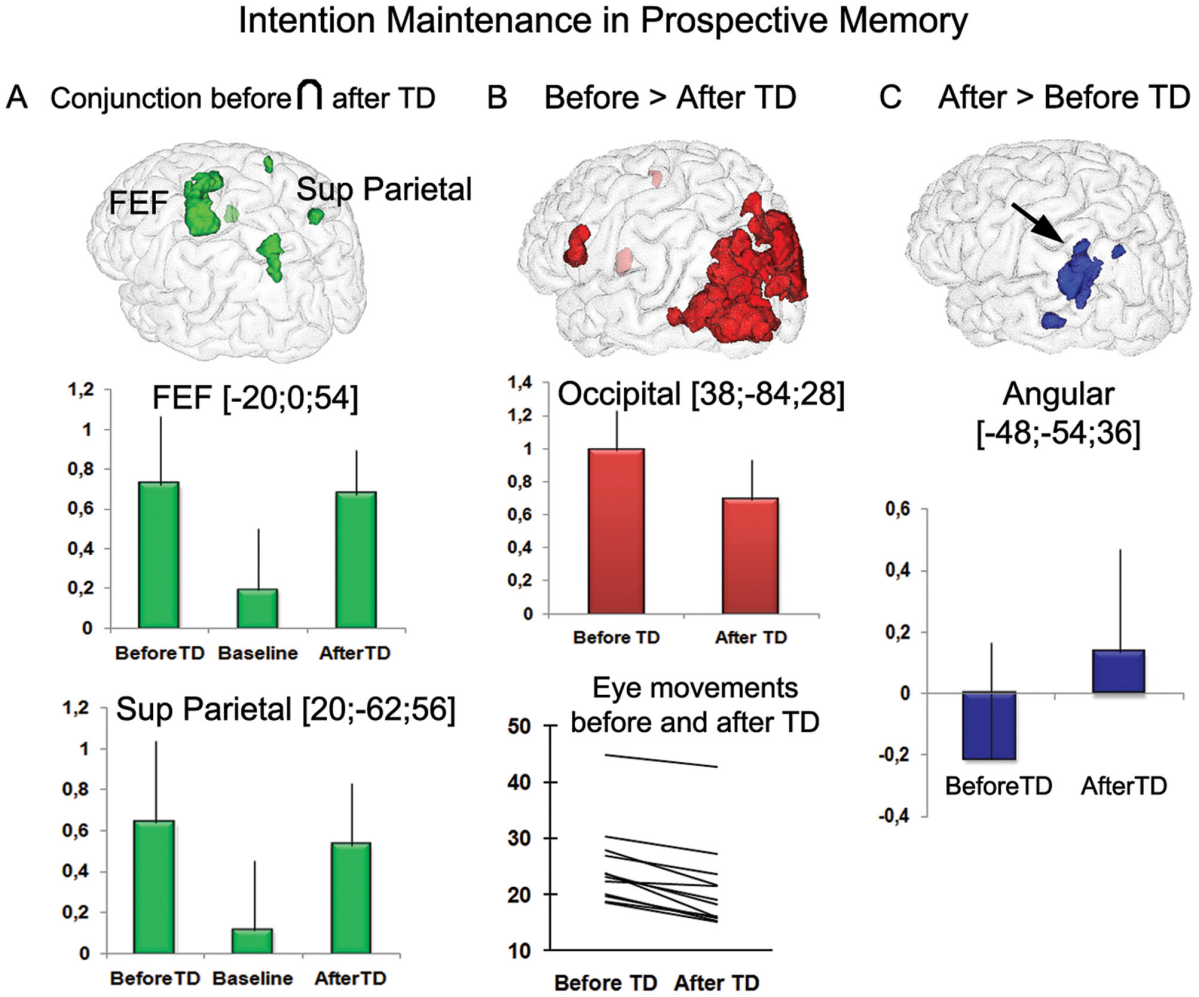
Similarities and differences in brain activity during the two intention maintenance phases. (A) A conjunction analysis showed that both the frontal eye fields (a) and the superior parietal cortex (b) were activated during the two intention maintenance phases (peak coordinates for activation plots correspond to those from the conjunction analysis). The comparison between these two phases showed that (B) the occipital cortex was more activated before than after TD (subpeak of the first cluster displayed in Table S2), and the analysis of the eye-tracking data demonstrated that the gaze covered more distance before than after TD (arbitrary units, independent of time duration); (C) the left inferior parietal cortex (angular gyrus) was more strongly activity after than before TD (subpeak of the second cluster displayed in Table S2). Activations, displayed at p<.005 for illustrative purposes, are overlaid on a 3-dimensional view of the MNI template. The Y-axis of the graphs (A) and (C) represents beta values.

The eye-tracking data, available for 12 subjects, allowed us to further analyze the distance covered by the gaze during intention maintenance before and after TD (Fig. 2). The results showed that all subjects covered more distance with their eyes before than after TD, regardless of time duration [paired t-test t(11) = 7.3, *p*<.0001], reflecting a more exploratory visual-search behavior before TD.

### Neuroimaging results

We addressed both similarities and differences between the two intention maintenance periods, occurring before and after TD. In order to reveal brain areas activated during both periods, we performed a conjunction analysis across the two intention maintenance phases that were initially contrasted with the “roaming” and “animation” phases (see Methods). This analysis revealed sustained activity of the frontal eye fields (FEF) and the superior parietal cortex (Fig. 2A, Table S2). These findings were further confirmed by the main effects of the two intention maintenance phases, where activity was found in these regions (Table S2).

The direct contrast between intention maintenance before and after TD showed a stronger activation of the occipital cortex before TD (Fig. 2B, Table S2). As suggested by the eye-tracking results reported above, this was likely caused by exploratory visual search before TD, in response to the goal of detecting a specific target in the environment.

After TD, sustained activity was more pronounced in the left inferior parietal cortex (angular gyrus). At p<.005, additional activity was found in the left intraparietal sulcus (Fig. 2C, Table S2).

**Figure 3:**
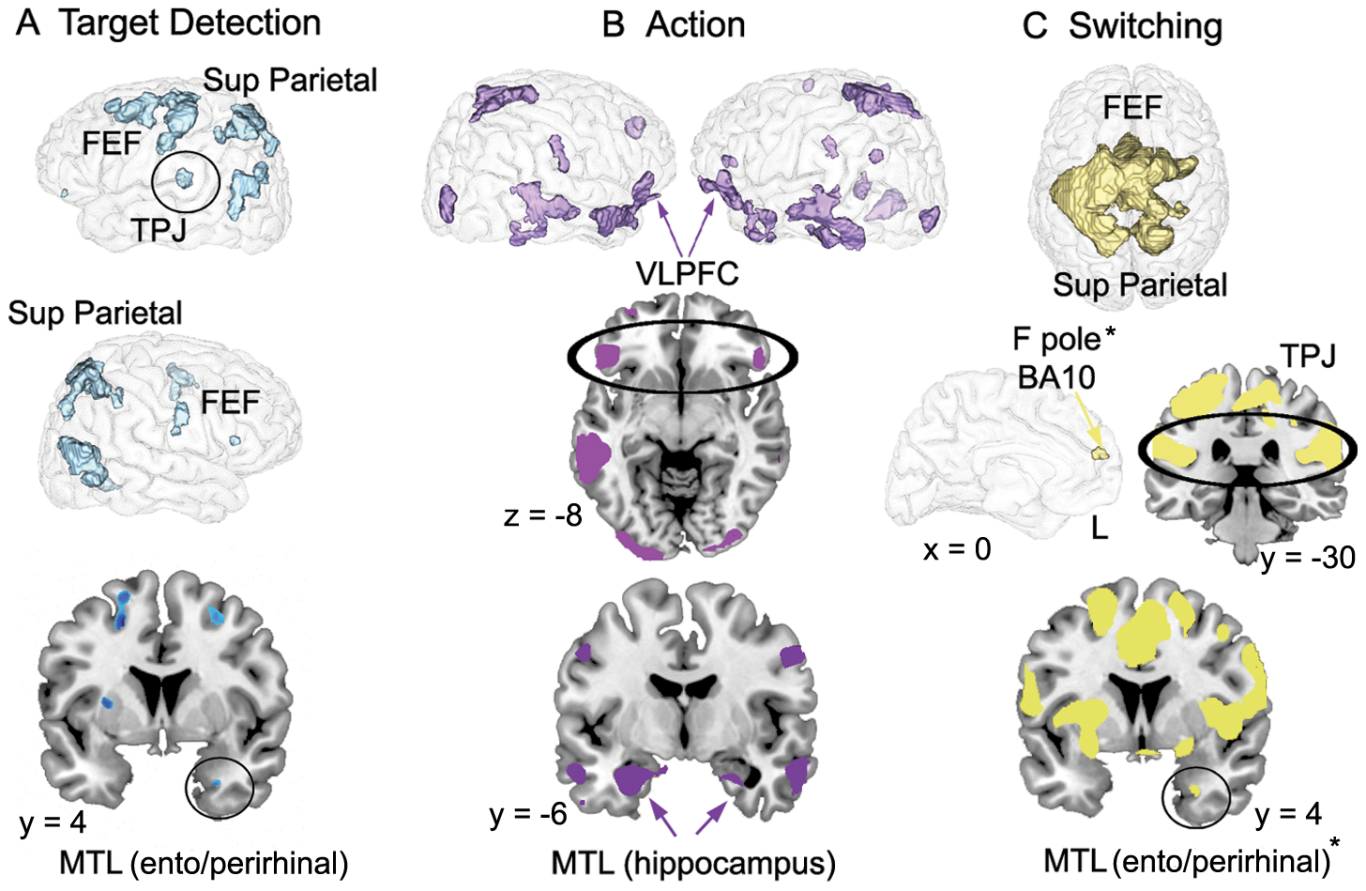
Brain areas activated at TD, action, and switching in comparison with their respective baselines. Notes: FEF = Frontal eye fields; TPJ = Temporo-parietal junction; MTL = Medial temporal lobe; VLPFC = Ventrolateral prefrontal cortex; F pole = Frontal pole. Activations are overlaid on a 3-dimensional view and sections of the MNI template. *These activations were significant at p<.005 but not significant at p<.001.

The two intention maintenance phases were dissociated by TD, which was associated with increased transient activity in the left temporo-parietal junction (TPJ), occipital areas, FEF and superior parietal cortex. A small cluster of activity was found in the right entorhinal cortex (Fig. 3A, Table S2).

At action, transient activity was observed in several regions of the brain, notably in the ventrolateral prefrontal cortex and the MTL, including the hippocampus with a left-sided preference (Fig. 3B, Table S2). Since the action phase corresponds to the retrospective component of PM, brain areas linked to episodic memory, such as prefrontal regions and the hippocampus, were expected [23].

Finally, we analyzed the neuroimaging data corresponding to the switching phase. The switching phase was defined as the time point (event) in between two PM tasks, where the previous task ended (end of an animation) and when a new intention would likely be activated. Switching was characterized by transient activity in the dorsal attentional system (FEF, superior parietal cortex) and ventral areas notably the TPJ (Fig. 3C, Table S2). At p<.005, additional activity was found in the right entorhinal cortex (Table S2). Frontopolar activity (BA 10), which was strongly expected in the present experiment, was revealed at p<.005 but was not significant at p<.001.

## Discussion

**Figure 4:**
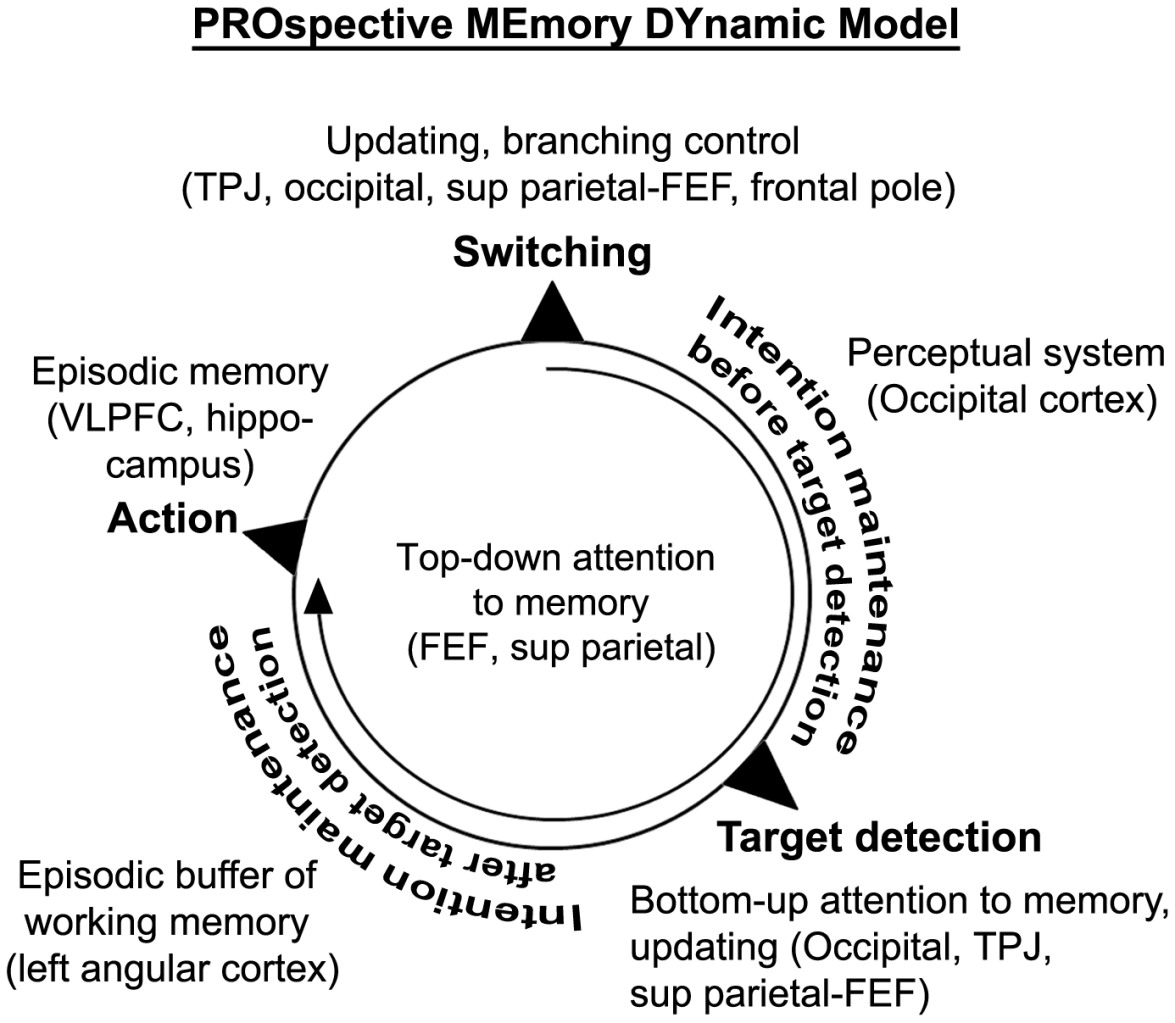
PROMEDY (PROspective MEmory DYnamic) model. The multi-phase iterative loop represents the phases involved while performing a PM task. To each phase we assigned one or more cognitive systems with their associated brain regions. The present experiment revealed that most of the PM intentions were self-initiated, resulting in an active intention maintenance phase before target detection; however it should be noted that a few intentions were triggered by the perception of the targets, characterized then by the absence of the first intention maintenance period.

A VR-based PM task was developed to approximate real-world experiences and allowed a more comprehensive examination of PM compared with the laboratory tasks used in previous neuroimaging research. Importantly, eye-tracking and post-scanning verbal assessment proved decisive in decomposing PM into subcomponents. We propose a neurocognitive model of PM, the PROspective MEmory DYnamic (PROMEDY) model, shaped as a multi-phase iterative loop, consisting of two intention maintenance phases, TD, action and switching (Fig. 4), with these phases involving an interactive engagement of perceptual, attentional and mnemonic networks as well as updating as developed below.

For most of the PM tasks, when the subjects made the decision to accomplish a PM task, the goal was actively maintained in mind until the effective realization of that task. Surprisingly, we did not find the typical frontopolar PFC (BA 10) engagement as in previous laboratory-based PM experiments, supposed to underpin intention maintenance [12] (see also discussion below). Instead we revealed the activation of an attentional network primarily shown in experiments on attention: the visual top-down attentional network (or dorsal system) [24] in which the FEF and the superior parietal cortex constitute key regions (see Fig. 2A and Table S2, conjunction analysis). As recently suggested, this network would also be engaged when attention is directed towards episodic memory retrieval (or attention to memory - AtoM) to support maintenance of goals in mind [25]. Thus, top-down attentional mechanisms would govern PM during almost the entire task until the action can be realized, while other additional neurocognitive systems were engaged, separately during each intention maintenance period.

Indeed, when comparing the two maintenance periods, it appeared that a perceptual (visual) system was more engaged before the target of interest was found, and inferior parietal areas were more activated in-between TD and action (Fig. 2B and 2C, Table S2, comparisons between the two intention maintenance phases). While the differential activation of the occipital areas is most likely linked to the search of the target (supported by the eye movement data, see Fig. 2B), the involvement of the inferior parietal cortex after TD was less expected. The function of this region is an ongoing matter of disagreement, and at least three different views have been suggested [26]. First, this parietal region could have a role in bottom-up AtoM. In contrast with top-down AtoM, where internal attention is directed towards the external world, attention is captured by an external cue that matches with the mental representation of what is to be recovered [25]. Second, it has been claimed that this region would subserve the episodic buffer sub-system of working memory, defined as a system able to temporarily maintain bound episodic information in working memory [27]. Third, this region may play the role of a mnemonic accumulator, such that it accumulates evidence until a criterion for the decision-making of recognition is reached [28]. A point that has been largely neglected in this debate is the exact location, within the inferior parietal cortex, of the three suggested systems. The fact that the angular gyrus and, less strongly, the intraparietal cortex were activated in a sustained manner between TD and action indicates that they may serve as memory buffers and/or accumulators rather than bottom-up AtoM, the latter being more likely to intervene in a transient manner. Regarding the episodic buffer hypothesis, a meta-analysis [29] showed that it is partly located in the angular gyrus. Concerning the mnemonic accumulator hypothesis, a review of the literature showed evidence that the intraparietal sulcus was mainly implicated [28]. Thus, the episodic buffer, underpinned by the angular gyrus, would mainly be involved during intention maintenance after TD, allowing maintenance of episodic information in relation to the target until the action can be performed. Although the activity of the intraparietal sulcus was revealed at a lenient threshold and would thus need further investigation, its function of information accumulation would fit well within the present construct of PM.

**Figure 5:**
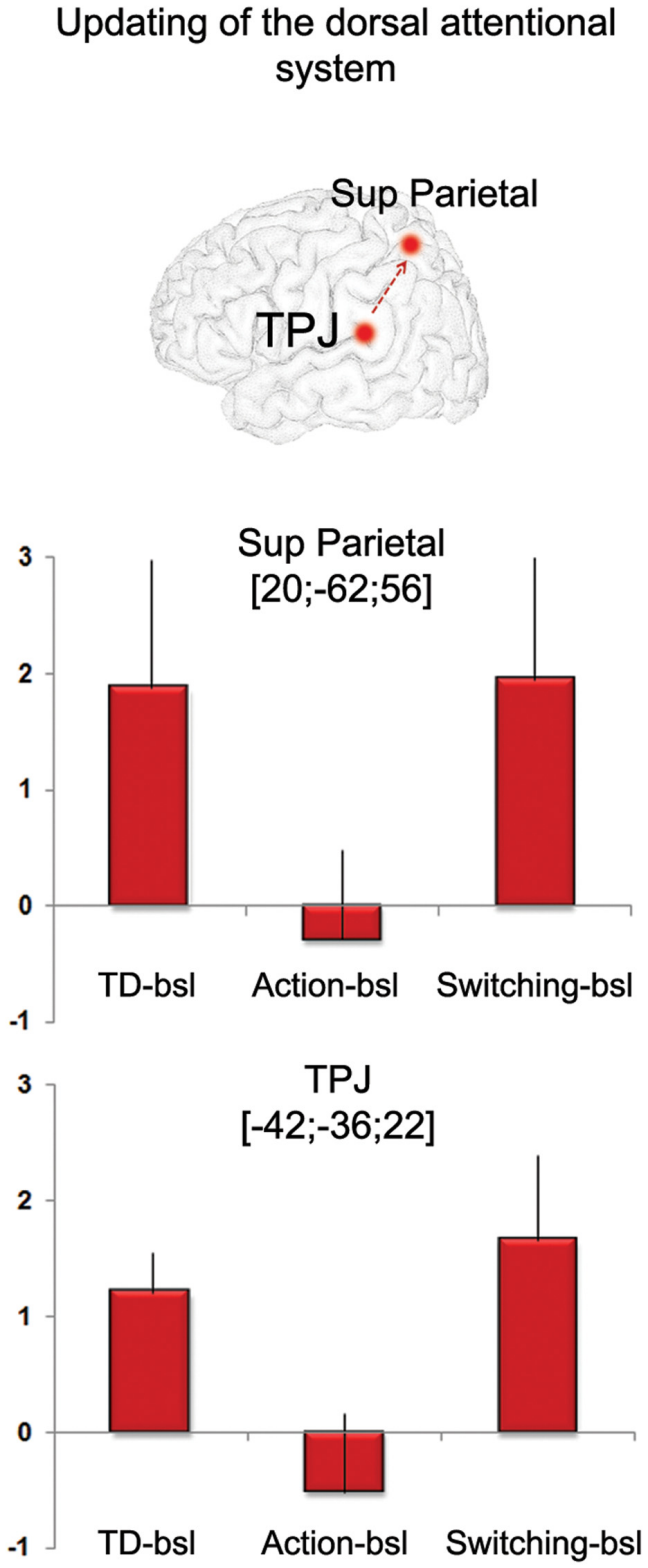
Activity of the temporo-parietal junction and superior parietal cortex. Peak coordinates corresponding to the temporo-parietal junction were taken from the contrast “TD versus End events”. Peak coordinates corresponding to the superior parietal cortex were taken from the conjunction analysis. In both ROIs, activity was significantly higher at TD and switching than action (paired t-tests, superior parietal regions: TD versus action t(11) = 2.48, *p* = .03, and switching versus action t(13) = 2.25, *p* = .04; TPJ: TD versus action t(11) = 3.02, *p* = .01, and switching versus action t(13) = 4.21, *p* = .001). The Y-axis represents the difference in activity (measured in beta values) between the condition of interest and its baseline.

This shift between the perceptual and mnemonic-like systems occurred when the target of interest was recognized, as determined by both the eye-tracking system and the verbal protocol. The most significant activity at TD appeared in the left TPJ. The occipital cortex was also activated, and less strongly the entorhinal/perirhinal cortex (see Fig. 3A, Table S2, contrast between TD and End events). At this time point, one can reasonably argue that the recognition of the target is based on the direct and automatic match between the mental set of what has to be retrieved and the present information in the environment. This cognitive mechanism corresponds to the definition of bottom-up AtoM, where information is transmitted from the occipital cortex and the MTL to the inferior parietal cortex [25]. While the AtoM model did not specify any precise location of bottom-up mechanisms within the inferior parietal lobe, according to the attentional theory [24], the TPJ is supposed to respond when a target is detected, although that theory suggests strong lateralization to the right hemisphere. In contrast, a meta-analysis revealed that this structure is related to successful episodic retrieval, but in the left hemisphere [30]. Thus, our findings corroborate this distinction between the attentional and AtoM models, with a process-based hemispheric asymmetry where the left TPJ would be engaged in attention directed toward memory [31]. In addition, at this time-point transient activation was observed in the FEF and the superior parietal cortex (Fig. 3A, Table S2), indicating interactions between the dorsal and ventral systems, where the goal-content of the dorsal system would be modulated via an updating mechanism supported by the TPJ [24], [25], [32], likely signaling that a part of the goal (find the target) has been accomplished (Fig. 5).

When the subjects had detected and arrived close enough to the target, they were enabled to execute the task. Despite the fact that the action phase was reduced to a single button press (a feature of the task that should be improved in future experiments), brain areas known to be involved in episodic memory retrieval were activated, notably the ventrolateral prefrontal cortex and regions of the medial temporal lobe (MTL) including the hippocampus (see Fig. 3B and Table S2, contrast between Action and Planning offset) [23]. The hippocampus has been strongly related to spatial memory, in humans and non-humans. Cellular recordings in the hippocampus in relation to spatial memory while rodents are freely moving in natural-like environments such as mazes have revealed insights into complex brain-behavior relations [33], [34]. VR studies in epileptic patients along with cellular recordings showed hippocampal neuronal firing for specific places [35]. We, too, observed hippocampal activity when subjects were located at specific places, but the current PM model indicates that in humans, hippocampal activity does not only reflect spatial information processing but the use of such information for episodic retrieval [36]. Activity in the hippocampus was mainly left-sided, which may indicate and further support the dissociation between the left *episodic* hippocampus and right *spatial* hippocampus [37]. The assumption that episodic memory was involved at Action is further supported by the fact that in the encoding phase, the subjects had to visualize themselves performing the tasks, elaborating then a representation for each action that was likely re-activated in the PM-VR experiment, and more particularly at Action.

In the neuroimaging literature, switching (or shifting) has been the second most present process together with intention maintenance in non-naturalistic PM experiments [38]. Indeed, in such tasks, and in contrast to the present experiment, PM is structured as a task to do *instead of* another task (the so-called *ongoing task*) and where the subjects have to immediately inhibit the ongoing activity in order to respond to a predefined prospective cue when it appears, resulting in i) the absence of an intention maintenance phase in between TD and action that is however frequent in real life, ii) the possible confound of target detection, execution and switching mechanism *per se*, which has made difficult the interpretation of BA 10 involvement in PM. In the current task (and most real-world PM tasks), switching is one phase of PM that makes possible the deactivation of a just-performed task and the activation of a new intention in mind. Interestingly, this was the only time point of the task where a frontopolar (BA 10) area was detected, however at a lenient threshold, which needs to be confirmed with other experiments (see Fig. 3C and Table S2, contrast between Switching and End events). Its hypothesized presence would nonetheless be supportive of its specific role in “branching” control, allowing attention shifting between tasks [39]. The TPJ and superior parietal cortex were also activated at switching (Fig. 3C and Table S2), indicating an updating process as for TD (Fig. 5), such that information that the task has been successfully accomplished is transmitted to the dorsal system, updating its content and likely inducing the activation of a new goal.

The combination of virtual reality fMRI, eye-tracking, and post-scanning verbal assessment were decisive in elaborating a more complete and realistic neuro-functional model of PM, PROMEDY (Fig. 4), defined as a multi-phase iterative loop with engagement of top-down attention throughout one PM task, allowing maintenance of a goal in mind, and a shift between perceptual processing (visual search) and mnemonic systems (episodic memory, episodic buffer in working memory) when the target was recognized. As for naturalistic studies of retrospective episodic memory (autobiographical memory), spatial navigation, and future thinking, using a combination of methods allowed us to contribute to the understanding of how the brain dynamically guides PM functioning in real-life. The proposed model may constitute a basis for future studies on normal and impaired PM, as we believe it can be applied to most real-world activity-based prospective events. This model is flexible and is prone to adjustments and improvements.

## Supporting Information

Table S1 Subject-by-subject behavioral results.
(0.07 MB DOC)

Table S2 Activations during the PM phases.
(0.17 MB DOC)

Movie S1 Overlay of 1) the VR environment in which a subject is performing two PM tasks (“Check the restaurant Rost”, and “Buy a hotdog”), 2) the eye-tracking data (yellow dot; the movie has intentionally been suspended for 5 seconds at both target detections), 3) the verbal reports from the subject in Swedish (subtitled in English), and 4) a movie of the brain areas activated at each PM phase (FEF = frontal eye fields, F pole = frontal pole (BA 10), HCP = hippocampus, IPS = intra-parietal sulcus, Lat Temp. = lateral temporal cortex, Occ. = occipital cortex, Paracing. = paracingulate cortex, Sup Par. = superior parietal cortex, TPJ = temporo-parietal junction, VLPFC = ventrolateral prefrontal cortex).
(12.92 MB MOV)
