# Supplementary material for: Neurocognitive Systems Related to Real-World Prospective Memory
Source: PLoS One. 2010 Oct 8;5(10):e13304. doi: 10.1371/journal.pone.0013304 (PMC2951914; doi:10.1371/journal.pone.0013304)
Supplement: Table S1 — Subject-by-subject behavioral results. (0.07 MB DOC) [file pone.0013304.s001.doc]

Table S1. Subject-by-subject behavioral results.

|  | Behavioral data | | Neuroimaging analyses | | | | | | | | | | | |
| --- | --- | --- | --- | --- | --- | --- | --- | --- | --- | --- | --- | --- | --- | --- |
|  | N exe actions | N Misses | Routes | IM before TD | | IM after TD | | TD | Action | Switching | Full PM Loops | | Half PM Loops | |
|  |  |  | N | N ev | tim dur (SD) | N ev | tim dur (SD) | N ev | N ev | N ev | N ev | tim dur (SD) | N ev | tim dur (SD) |
| S01 | 22 | 0 | 4 | 9 | 18.2 (9) | 18 | 8.4 (3.7) | 18 | 18 | 14 | 9 | 33.6 (9.2) | 8 | 15.5 (4.4) |
| S02 | 22 | 0 | 3 | 11 | 10.9 (11.3) | 13 | 8.7 (4.5) | 13 | 22 | 17 | 11 | 27.7 (11) | 1 | 9.6 (-) |
| S03 | 22 | 0 | 5 | 14 | 11.8 (11.8) | 22 | 12.3 (6.5) | 22 | 22 | 17 | 14 | 30.8 (10.6) | 4 | 19.8 (8.7) |
| S04 | 20 | fo. (1), fa. (1) | 5 | - | - | - | - | - | 17 | 14 | - | - | - | - |
| S05 | 22 | 0 | 5 | 16 | 17.9 (14.1) | 22 | 11.7 (6.6) | 22 | 22 | 17 | 16 | 36.9 (13) | 3 | 21.2 (2.3) |
| S06 | 20 | fo. (1), fa. (1) | 5 | - | - | - | - | - | 20 | 17 | - | - | - | - |
| S07 | 21 | fo. (1) | 5 | 15 | 20.3 (14.1) | 21 | 15.2 (9.2) | 21 | 21 | 16 | 15 | 43.3 (16.3) | 4 | 21.3 (8.6) |
| S08 | 22 | 0 | 5 | 13 | 12.9 (12.2) | 22 | 10.9 (6.6) | 22 | 22 | 17 | 13 | 31.6 (13.3) | 8 | 18.2 (4.1) |
| S09 | 22 | 0 | 5 | 17 | 13.4 (11.4) | 22 | 11.1 (5.5) | 22 | 22 | 17 | 17 | 31.8 (11.9) | 4 | 21 (7) |
| S10 | 22 | 0 | 5 | 15 | 15.5 (12.3) | 21 | 10 (3.8) | 21 | 22 | 17 | 15 | 33.8 (11.4) | 2 | 14.6 (7.5) |
| S11 | 21 | fa. (1) | 5 | 16 | 17 (10.7) | 21 | 15.3 (9.8) | 21 | 21 | 17 | 16 | 38.4 (10.4) | 4 | 29.7 (18.3) |
| S12 | 21 | fa. (1) | 5 | 13 | 16.9 (12.6) | 21 | 9.9 (5.9) | 21 | 21 | 17 | 13 | 34.2 (8.6) | 6 | 15.9 (5.3) |
| S13 | 22 | 0 | 5 | 14 | 20.1 (15.8) | 22 | 10.3 (6.3) | 22 | 22 | 17 | 14 | 37.1 (16.2) | 7 | 19.9 (8) |
| S14 | 23 | rep. act. (1) | 5 | 17 | 12.9 (11.1) | 23 | 12.7 (7.2) | 23 | 23 | 19 | 17 | 32.6 (11.6) | 5 | 20.3 (10.2) |
| mean | 21.6 |  |  | 14.2 | 15.7 (12.2) | 20.7 | 11.4 (6.3) | 17.7 | 21.1 | 16.6 | 14.2 | 34.3 (12) | 4.7 | 18.9 (7.7) |
| SD | 0.8 |  |  | 2.4 | 3.21 (1.9) | 2.7 | 2.2 (1.9) | 7.9 | 1.7 | 1.3 | 2.4 | 4.1 (2.4) | 2.2 | 4.9 (4.2) |

Notes: IM: Intention maintenance, N ev: Number of events, SD: Standard Deviation, TD: Target Detection, tim dur: time duration in seconds. Fo. (Forgotten) means that a PM task was totally forgotten to be performed; fa. (Failed) means that the subject was not able to find the place of a specific action; the rep. act. (Repeated action) refers to the "glasses" task where the subject S14 checked the price of a pair of glasses twice, at two different optician stores. The last PM task of each route was not considered as Switching but rather as the End of the route since no further PM task was to be performed, except for S14 who looked forward another action to perform at the end of a route. The column "Full Loops" refers to the PM tasks where the intention was self-initiated; Time duration includes 1) IM before TD, 2) IM after TD, 3) animation. The column "Half Loops" refers to the PM tasks where the intention was triggered by the perception of the target (no intention maintenance before TD); Time duration includes 1) IM after TD, 2) animation.
