# Supplementary material for: Neurocognitive Systems Related to Real-World Prospective Memory
Source: PLoS One. 2010 Oct 8;5(10):e13304. doi: 10.1371/journal.pone.0013304 (PMC2951914; doi:10.1371/journal.pone.0013304)
Supplement: Table S2 — Activations during the PM phases. (0.17 MB DOC) [file pone.0013304.s002.doc]

Table 2. Activations during the PM phases.

| Anatomical localization | BA | Hs | x | y | z | T | k (p.001) | k (p.005) |
| --- | --- | --- | --- | --- | --- | --- | --- | --- |
| Intention Maintenance before TD > Baselines (Roaming + Animation) | | | | | | | | |
| Parietal sup, Occipital sup | 7/19 | L | -22 | -76 | 44 | 7.65 | 259 | 697 |
| Frontal sup/mid (FEF) | 6/8 | R | 22 | 8 | 60 | 7.48 | 153 | 500 |
| Vermis |  | M | 0 | -62 | -24 | 6.97 | 32 | 96 |
| Cingulate mid, SMA | 6 | L | -12 | -4 | 50 | 6.72 | 101 | 1482 |
| Parietal sup | 7 | R | 20 | -64 | 58 | 6.35 | 105 | 582 |
| Sup Frontal sulcus/FEF | 6/8 | L | -20 | 2 | 54 | 6.11 | 112 | subpeak |
| Precentral | 6 | L | -34 | -14 | 52 | 5.88 | 191 | subpeak |
| Occipital mid | 19 | L | -32 | -84 | 18 | 5.45 | 28 | 147 |
| Cingulate mid | 23 | L | -14 | -20 | 42 | 5.27 | 13 | subpeak |
| Occipital mid | 19 | R | 36 | -82 | 16 | 5.04 | 30 | 273 |
| Cerebellum |  | R | 20 | -44 | -24 | 4.97 | 9 | 136 |
| Cerebellum |  | L | -18 | -48 | -16 | 4.89 | 4 | 29 |
| Parietal sup | 7 | R | 20 | -72 | 50 | 4.72 | 19 | subpeak |
| Intention Maintenance after TD > Baselines (Roaming + Animation) | | | | | | | | |
| Precentral, postcentral | 6/4 | R | 36 | -14 | 60 | 6.63 | 142 | 476 |
| Frontal sup, SMA, pre/postcentral (FEF) | 6/4 | L | -38 | -20 | 56 | 6.30 | 442 | 1400 |
| Parietal sup, occipital sup | 7 | L | -20 | -60 | 56 | 6.22 | 45 | 345 |
| Parietal inf | 40 | L | -48 | -46 | 42 | 6.17 | 12 | 32 |
| Vermis |  | R | 4 | -50 | -22 | 5.71 | 7 | 29 |
| Insula | 48 | R | 44 | 2 | -2 | 5.52 | 11 | 28 |
| Sup Frontal sulcus/FEF | 6 | L | -20 | 0 | 56 | 5.48 | 32 | subpeak |
| SMA | 6 | L | -4 | 0 | 60 | 4.99 | 18 | 227 |
| Occipital sup | 7 | L | -22 | -72 | 36 | 4.69 | 5 | subpeak |
| Cerebellum |  | R | 28 | -42 | -34 | 4.68 | 2 | 95 |
| Parietal sup | 7 | R | 20 | -62 | 56 | 4.51 | 3 | 57 |
| Conjunction Intention maintenance before ∩ after target detection | | | | | | | | |
| Pre/postcentral | 6/4 | L | -34 | -14 | 54 | 5.37 | 114 | 725 |
| Sup Frontal sulcus/FEF | 6 | L | -20 | 0 | 54 | 4.84 | 12 | subpeak |
| SEF | 6 | L | -4 | 2 | 58 | 4.74 | 5 | 143 |
| Occipital Sup | 7 | L | -22 | -72 | 36 | 4.69 | 5 | 169 |
| Parietal Sup | 7 | R | 20 | -62 | 56 | 4.51 | 3 | 55 |
| Parietal Sup | 7 | L | -22 | -62 | 52 | 4.51 | 2 | subpeak |
| Frontal sup (FEF)* | 6 | R | 32 | -12 | 60 | 3.98 | NS | 21 |
| Intention maintenance before > after target detection | | | | | | | | |
| Lingual,calcarine, precuneus | 18/19/30 | R | 20 | -48 | -10 | 7.82 | 1074 | 6944 |
| Lingual | 18 | R | 10 | -74 | -6 | 7.61 | 203 | subpeak |
| Lingual/Calcarine | 17/18/19 | L | -6 | -90 | -2 | 7.40 | 779 | subpeak |
| Occipital sup/Parietal sup | 7 | R | 26 | -78 | 50 | 6.76 | 280 | subpeak |
| Fusiform | 20 | R | 36 | -8 | -34 | 6.46 | 16 | 44 |
| Occipital sup | 19 | L | -20 | -82 | 38 | 6.02 | 161 | subpeak |
| Frontal ventrolateral | 47 | R | 26 | 30 | -8 | 5.83 | 23 | 68 |
| Insula, Frontal inf | 48/45/47 | L | -36 | 24 | 6 | 5.73 | 36 | 248 |
| Temporal mid | 21 | R | 42 | -44 | 10 | 4.55 | 5 | 34 |
| Occipital sup | 19 | L | -18 | -68 | 40 | 4.41 | 16 | subpeak |
| Intention maintenance after > before target detection | | | | | | | | |
| Temporal mid | 37 | L | -58 | -54 | -6 | 5.14 | 24 | 87 |
| Angular, temporal mid | 39/21/22 | L | -46 | -52 | 24 | 5.08 | 110 | 649 |
| Occipital inf | 19 | L | -40 | -82 | -8 | 4.91 | 4 | 13 |
| Intraparietal sulcus* | 7/39 | L | -40 | -74 | 46 | 3.77 | NS | 17 |
| Target detection > Baseline (End events) | | | | | | | | |
| TPJ | 41 | L | -42 | -36 | 22 | 7.95 | 36 | 79 |
| Frontal mid/precentral (FEF) | 6 | L | -20 | -18 | 66 | 7.57 | 313 | 1289 |
| Parietal sup, precuneus | 7/5 | R | 8 | -62 | 66 | 7.36 | 98 | 943 |
| Occipital mid, temporal mid | 39/37 | R | 42 | -80 | 28 | 7.12 | 167 | 603 |
| Occipital mid | 39/19 | L | -40 | -82 | 28 | 6.75 | 183 | 486 |
| Sup Frontal sulcus/FEF | 8 | L | -26 | 14 | 54 | 5.55 | 96 | subpeak |
| Precuneus/Parietal sup | 5/7 | R | 14 | -50 | 56 | 5.54 | 78 | subpeak |
| Lingual | 18 | R | 18 | -64 | -8 | 5.33 | 28 | 88 |
| Occipital mid | 19 | L | -32 | -84 | 38 | 5.32 | 24 | subpeak |
| Precuneus/Parietal sup | 5/7 | L | -6 | -56 | 66 | 5.19 | 46 | subpeak |
| Precentral (FEF) | 6 | R | 40 | -4 | 44 | 4.84 | 6 | 205 |
| Temporal mid | 37 | R | 52 | -70 | 2 | 4.68 | 16 | subpeak |
| Putamen |  | L | -30 | 2 | 6 | 4.52 | 4 | 25 |
| Precuneus | 7 | L | -14 | -68 | 62 | 4.47 | 8 | subpeak |
| Parietal sup | 5 | R | 20 | -52 | 66 | 4.37 | 4 | subpeak |
| Ento/perirhinal | 36 | R | 28 | 6 | -34 | 4.18 | 1 | 7 |
| Action > Baseline (Planning offset) | | | | | | | | |
| Temporal mid | 21 | R | 62 | -6 | -18 | 7.87 | 120 | 730 |
| Temporal inf, fusiform | 21/37 | L | -46 | -46 | -24 | 7.66 | 82 | 1213 |
| Paracentral lobule, precuneus | 4/5 | LR | -2 | -30 | 72 | 7.24 | 394 | 922 |
| Cuneus | 17 | R | 10 | -100 | 10 | 7.15 | 166 | 289 |
| Postcentral | 3/4 | R | 56 | -4 | 38 | 6.57 | 48 | 123 |
| Paracingulate | 32 | R | 14 | 50 | 24 | 6.19 | 58 | 123 |
| Temporal pole sup | 38 | R | 38 | 20 | -28 | 5.75 | 32 | subpeak |
| Temporal pole mid | 20 | R | 46 | 8 | -30 | 5.74 | 55 | subpeak |
| Temporal mid | 21 | R | 66 | -40 | -2 | 5.74 | 53 | 421 |
| Temporal mid | 21 | L | -58 | -42 | -4 | 5.62 | 224 | subpeak |
| Hippocampus (head), entorhinal, amygdala | 36 | L | -32 | 0 | -28 | 5.56 | 91 | 290 |
| Frontal inf orb | 47 | R | 52 | 24 | -10 | 5.48 | 39 | subpeak |
| Lingual | 17/18 | LR | 0 | -72 | 6 | 5.37 | 102 | 326 |
| Occipital inf | 19 | L | -48 | -70 | -18 | 5.13 | 72 | subpeak |
| Temporal mid | 21 | L | -52 | 10 | -22 | 5.01 | 9 | 201 |
| Temporal mid | 20 | R | 50 | -30 | -10 | 4.93 | 24 | 86 |
| Occipital mid | 17 | L | -14 | -106 | 4 | 4.86 | 39 | 138 |
| Temporal mid | 37 | R | 62 | -58 | 12 | 4.84 | 42 | subpeak |
| Frontal ventrolateral | 38 | L | -34 | 24 | -24 | 4.59 | 16 | 359 |
| Frontal ventrolateral | 47 | L | -48 | 46 | -16 | 4.52 | 13 | subpeak |
| Cerebellum |  | R | 26 | -30 | -36 | 4.43 | 9 | 77 |
| Frontal ventrolateral | 38 | L | -44 | 26 | -14 | 4.39 | 4 | subpeak |
| Precentral | 6 | R | 32 | -26 | 62 | 4.36 | 7 | 77 |
| Cerebellum |  | R | 24 | -70 | -30 | 4.29 | 3 | 131 |
| Supramarginal | 40 | R | 66 | -32 | 38 | 4.21 | 6 | 102 |
| Hippocampus/Entorhinal | 28 | R | 20 | -4 | -24 | 3.97 | 1 | 69 |
| Switching > Baseline (End events) | | | | | | | | |
| Pre/postcentral, SFS/FEF/SEF, Ant cingulate | 3/ 4/6/24 | LR | -32 | -12 | 54 | 11.33 | 5925 | 10870 |
| Cuneus, occipital sup | 18/19 | R | 12 | -80 | 30 | 9.48 | 139 | 360 |
| TPJ, smg, Temporal sup, Rol oper | 41/42/48 | L | -48 | -22 | 18 | 9.17 | 678 | 1336 |
| TPJ, smg, Temporal sup, Rol oper | 41/42/48 | R | 66 | -40 | 28 | 7.83 | 842 | 2718 |
| Temporal and occipital mid | 37/19/39 | R | 48 | -68 | 0 | 7.09 | 701 | 1119 |
| Parietal sup, precuneus | 5/7 | R | 10 | -50 | 52 | 6.89 | 697 | subpeak |
| Precentral/FEF (BA 6) | 6 | R | 34 | -4 | 42 | 6.47 | 159 | subpeak |
| Putamen |  | L | -26 | 4 | 6 | 5.5 | 232 | 1012 |
| Occipital mid | 19 | L | -42 | -84 | 30 | 5.29 | 44 | 667 |
| Insula (BA 48) | 48 | R | 46 | 6 | 2 | 5.26 | 276 | subpeak |
| Rolandic operculum (BA 48) | 48 | R | 46 | -2 | 10 | 4.88 | 21 | subpeak |
| ventral striatum (putamen) |  | R | 20 | 8 | -12 | 4.73 | 24 | 193 |
| Occipital mid (BA 19) | 19 | L | -48 | -74 | 2 | 4.72 | 174 | subpeak |
| Cerebellum, lingual | 18 | R | 14 | -54 | -20 | 4.64 | 169 | 641 |
| Calcarine | 17 | L | -2 | -88 | -6 | 4.6 | 29 | 116 |
| Rolandic operculum (BA 48) |  | L | -56 | 2 | 12 | 4.08 | 22 | subpeak |
| Ento/perirhinal* | 35 | R | 26 | 4 | -34 | 3.77 | NS | 19 |
| Frontal pole* | 10 | L | -8 | 60 | 10 | 3.58 | NS | 35 |

Notes: The minimum T values corresponding to p < .001 are T = 4.29 (Intention maintenance before and after TD compared to roaming+animation), T=4.30 (conjunction between the 2 intention maintenance phases), T=4.02 (comparisons between the intention maintenance phases and target detection vs. end events), T=3.85 (action vs. planning offset and switching vs. end events). Abbreviations: ant = anterior, BA = Brodmann Area, FEF = Frontal Eye Field, Hs = Hemisphere, inf = inferior, k = cluster size (displayed for both p < .001 and p < .005), L = Left, med = medial, mid = middle, NS = No Significant, R = Right, rol oper = rolandic operculum, SEF = Supplementary Eye Field, smg = supramarginal, sup = superior, TPJ = Temporo-Parietal Junction. *Regions that were significant at p < .005 but not at p < .001.
